# Managing medication-related osteonecrosis of jaw: A prospective observational study

**DOI:** 10.6026/973206300214144

**Published:** 2025-11-15

**Authors:** Mahendra Kumar Dadarwal, Timbadiya Vijaykumar Mansukhbhai, Nishad Gawali, Pradeep Kumar Dohare, Sumit Bhatt, Swati Kharat, Rashmi Laddha

**Affiliations:** 1 Department of Oral Surgery, Rajasthan Dental College, Jaipur, Rajasthan, India; 2 Department of Oral Pathology and Microbiology, Rishiraj College of Dental Sciences and Research Centre, Bhopal, Madhya Pradesh, India; 3 Department of Public Health Dentistry, Hazaribag College of Dental Sciences and Hospitals, Hazaribagh, Jharkhand, India; 4 Department of Community Medicine, SRVS Medical College, Shivpuri, Madhya Pradesh, India; 5 Department of Oral and Maxillofacial Surgery, Rajasthan Dental College and Hospital, Jaipur, Rajasthan, India; 6 Department of Prosthodontics, Dr RR Kambe Dental College and Hospital, Akola, Maharashtra, India

**Keywords:** Medication-related osteonecrosis of the jaw (MRONJ), antiresorptive therapy, management protocols, jaw osteonecrosis, conservative treatment, surgical intervention, bone healing

## Abstract

Medication-related osteonecrosis of the jaw (MRONJ) remains a significant clinical challenge due to its complex etiology and variable response to treatment. This prospective observational study evaluated the effectiveness of current management protocols in 130 patients receiving antiresorptive or antiangiogenic therapies. Patients were assessed clinically and radiographically and treated according to standardized stage-specific guidelines. Combined conservative and surgical interventions achieved better healing rates, symptom control, and improved quality of life over 12 months. These findings support ongoing refinement of stage-based MRONJ protocols to optimize therapeutic outcomes and patient care.

## Background:

Medication-related osteonecrosis of the jaw (MRONJ) is a serious adverse effect associated primarily with antiresorptive agents such as bisphosphonates and denosumab, as well as certain antiangiogenic drugs used in oncology and osteoporosis management [1]. MRONJ is characterized by exposed necrotic bone in the maxillofacial region that fails to heal within eight weeks in patients with current or previous exposure to these medications, without prior radiation therapy to the jaws [2]. The condition can cause significant morbidity, including pain, infection and impaired oral function, severely affecting patients' quality of life [3]. The pathophysiology involves suppression of bone remodeling, inhibition of angiogenesis and potential immune dysfunction, which collectively impair bone healing and increase susceptibility to necrosis [4]. Management of MRONJ remains challenging, with treatment protocols evolving to balance conservative and surgical approaches based on disease stage and patient factors [5]. Early-stage lesions may respond to conservative measures such as antimicrobial rinses, antibiotics and pain management, while advanced stages often require surgical debridement or resection [6]. However, standardized treatment guidelines are still under refinement and clinical outcomes vary widely. Therefore, it is of interest to evaluate the effectiveness of current management protocols in a representative patient cohort, analyzing clinical outcomes, healing rates and factors influencing treatment success to provide evidence-based recommendations for optimizing MRONJ care.

## Materials and Methods:

This prospective observational study included 130 patients diagnosed with medication-related osteonecrosis of the jaw (MRONJ) from January 2023 to December 2024 at a tertiary care center. Eligible patients were adults (age ≥18 years) with a history of antiresorptive (bisphosphonates or denosumab) or antiangiogenic therapy and clinically and radiographically confirmed MRONJ, defined according to the American Association of Oral and Maxillofacial Surgeons (AAOMS) criteria. Patients with prior radiation therapy to the jaws or metastatic bone disease involving the maxillofacial region were excluded. All patients underwent comprehensive clinical evaluation including history, symptom assessment and staging of MRONJ based on AAOMS guidelines (Stage 0 to Stage 3). Radiographic assessment included panoramic radiographs and cone-beam computed tomography (CBCT) as needed. Treatment was tailored according to disease stage: conservative management for Stage 0 and Stage 1 cases involving antimicrobial mouth rinses, systemic antibiotics when indicated, analgesics and close monitoring; and surgical intervention for Stage 2 and Stage 3 patients involving sequestrectomy, debridement, or resection with adjunctive antimicrobial therapy. Patients were followed up at regular intervals over 12 months to assess symptom resolution, lesion healing, recurrence and quality of life using standardized questionnaires. Data on demographics, medication history, comorbidities, treatment modalities and outcomes were collected and analyzed. Statistical analysis was performed using descriptive and inferential methods, with significance set at p < 0.05.

## Results:

This study included 130 patients with MRONJ, with a mean age of 62.4 ± 10.8 years; 78 (60%) were female and 52 (40%) male. The majority (70%) had a history of bisphosphonate use, while 20% were on denosumab and 10% received antiangiogenic agents. MRONJ staging at presentation was: Stage 0 - 15 (11.5%), Stage 1 - 40 (30.8%), Stage 2 - 50 (38.5%) and Stage 3 - 25 (19.2%). The mandible was involved in 82% of cases, while 18% involved the maxilla. Table 1 (see PDF) shows Patients with early-stage MRONJ (Stages 0 and 1) primarily received conservative treatment, with a high rate of symptom control and lesion stabilization. Table 2 (see PDF) shows Symptom resolution and lesion healing rates improved significantly with appropriate stage-based management. Table 3 (see PDF) shows Quality of life scores improved markedly in patients who achieved lesion healing. Table 4 (see PDF) shows Mandibular involvement was associated with higher severity and need for surgical intervention. Table 5 (see PDF) shows Antiresorptive drug duration correlated with disease severity. Table 6 (see PDF) shows surgical intervention demonstrated higher healing rates in advanced-stage MRONJ. Table 7 (see PDF) shows Comorbidities such as diabetes and corticosteroid use were associated with poorer outcomes. Table 8 (see PDF) shows Patient adherence to follow-up and treatment protocol influenced success rates. Table 9 (see PDF) shows No significant difference in outcomes was observed between bisphosphonate and denosumab users. Table 10 (see PDF) shows early intervention was linked to better prognosis.

## Discussion:

This study assessed the current management protocols of medication-related osteonecrosis of the jaw (MRONJ) in a cohort of 130 patients, revealing important insights into treatment outcomes across different disease stages. Consistent with previous research, we observed that early-stage MRONJ (Stage 0 and 1) responds well to conservative management, including antimicrobial rinses and symptom control, with a high rate of lesion stabilization and symptom resolution. This reinforces the importance of early diagnosis and prompt conservative care to prevent disease progression. Patients with advanced-stage MRONJ (Stages 2 and 3) benefitted significantly from surgical intervention, which resulted in substantially higher complete healing rates compared to conservative treatment alone [7]. This supports current guidelines recommending tailored surgical debridement or resection in advanced cases to remove necrotic bone and promote healing [8]. The mandible was more frequently involved and associated with higher disease severity, which may relate to its dense cortical bone and reduced vascularity compared to the maxilla, consistent with previous findings [9]. The duration of antiresorptive therapy was positively correlated with MRONJ severity, with longer treatment durations predisposing patients to more advanced lesions [10]. This highlights the need for careful monitoring and preventive dental evaluations in patients receiving prolonged antiresorptive or antiangiogenic therapies. Additionally, comorbid conditions such as diabetes and corticosteroid use negatively impacted healing outcomes, likely due to compromised immune response and impaired tissue repair, underscoring the importance of optimizing systemic health during management [11]. Adherence to follow-up and treatment protocols was a key factor in successful outcomes, emphasizing the role of patient education and regular monitoring. Interestingly, no significant differences in healing rates were noted between bisphosphonate and denosumab users, suggesting that the risk and prognosis of MRONJ may be comparable between these agents [12]. Icariin benefits MRONJ in terms of the area of soft tissue wound and ratio of empty lacuna. Teriparatide activates expression of RANKL and reduces the area of bone necrosis and ratio of empty lacuna in a MRONJ lesion [13]. Early initiation of treatment following MRONJ diagnosis was strongly associated with better prognosis, further reinforcing the value of timely intervention. Limitations of this study include its observational design and single-center setting, which may limit generalizability. Nevertheless, these findings contribute valuable real-world data supporting stage-specific management strategies and highlight the need for multidisciplinary collaboration involving dental, medical and surgical teams to optimize care for MRONJ patients.

## Conclusion:

Medication-related osteonecrosis of the jaw poses a significant clinical challenge requiring stage-specific management. Early diagnosis and conservative treatment yield favorable outcomes in initial stages, while surgical intervention improves healing in advanced cases. Multidisciplinary care and patient adherence are essential for optimizing treatment success and quality of life.
